# Correlation of bilirubin and toxic bile acids in critically ill patients with cholestatic liver dysfunction and adsorber application

**DOI:** 10.1038/s41598-024-72676-6

**Published:** 2024-09-18

**Authors:** Caroline Gräfe, Helen Graf, Vassilissa Wustrow, Uwe Liebchen, Philippe Conter, Michael Paal, Katharina Habler, Christina Scharf

**Affiliations:** 1 Department of Anesthesiology, LMU University Hospital, LMU Munich, Marchioninistrasse 15, 81377, Munich, Germany; 2 Institute of Laboratory Medicine, LMU University Hospital, LMU Munich, Marchioninistrasse 15, 81377 Munich, Germany

**Keywords:** Biliary tract disease, Liver diseases

## Abstract

Bilirubin is one of the most frequently used laboratory values to monitor critically ill patients with cholestatic liver dysfunction. Besides bilirubin, toxic bile acids (TBAs), which may cause severe organ damage, are typically elevated. A correlation between both parameters seems plausible, but data are lacking. The aim was to investigate whether there is a correlation between bilirubin and TBAs in patients’ blood and whether a compareable reduction can be observed during the use of the adsorber CytoSorb (CS). As part of the Cyto-SOLVE study (NCT04913298), 16 critically ill patients with cholestatic liver dysfunction, bilirubin concentration > 10 mg/dl, continuous kidney replacement therapy and CS-application were investigated. Bilirubin and TBA concentrations were measured from arterial blood at defined time points (before start, after 6 and 12 h). Relative reduction (RR) was calculated using the formula$\:\:{\boldsymbol{RR}}\:{\boldsymbol{\left(\%\right)}}=\frac{{\boldsymbol{concentration\left(pre-post\right)}}}{{\boldsymbol{concentration\left(pre\right)}}}*{\boldsymbol{100}}$. A moderate to high correlation between bilirubin and TBA concentration at all defined timepoints (r_start_=0.64, *p* = 0.008; r_6h_ = 0.85, *p* < 0.001, r_12h_ = 0.72, *p* = 0.002) was observed. In the first six hours of CS-application, a significant elimination of TBA (median TBA: 30.8→20.1µmol/l, *p* < 0.001) and bilirubin (median bilirubin: 17.1→11.9 mg/dl, *p* < 0.001) was observed. The median RR after 6 h was 26.1% and 39.8% for bilirubin and TBA, respectively. No further reduction was observed after 12 h (RR_bilirubin_: – 0.6%, RR_TBA_: 1.8%). There was an at least moderate correlation between bilirubin and TBA in patients with cholestatic liver dysfunction. Therefore, bilirubin seems to be a suitable surrogate parameter for TBA elimination during CytoSorb application.

## Introduction

Cholestatic liver dysfunction frequently appears in intensive care units (ICU) due to various reasons such as sepsis, viral infections, multi-organ failure, and medication side effects^1^. Alongside traditional liver function indicators, there is a notable rise in bile acids (BA), particularly toxic bile acids, which further harm hepatocytes and other organ tissues^2,3^. Yet, decisions about treatments, like employing extracorporeal liver support systems, often hinge on bilirubin concentration. Notably, therapeutic strategies, like extracorporeal liver support systems, are often determined by bilirubin concentration — even though bilirubin poses minimal harm to adult humans^4^, and its correlation with more toxic indicators like TBA is rarely analysed^5^. To date, the existence of a correlation between bilirubin and TBA concentrations in patients with cholestatic liver dysfunction is unclear. One commonly used liver support device is the cytokine adsorber CytoSorb, which was originally developed to remove cytokines but has since been licensed for the removal of bilirubin^6^. This device employs porous polymer sorbent beads, which offer a surface area exceeding 45,000 square meters, effectively removing substances with molecular sizes up to 60 kDa^7^. It has not been investigated whether the rate of reduction by CS is comparable for both parameters. This study seeks to determine correlation between bilirubin and TBA concentrations in patients with cholestatic liver dysfunction and to evaluate the efficacy of CS therapy on these parameters.

## Methods

*Study setting*: This was a monocentric, prospective observational study investigating a potential correlation of bilirubin and TBA and the elimination of both during the application of the adsorber CS. Patients were included between May 2021 and August 2022 during their stay at two ICUs at the LMU university hospital in Munich. The local institutional review board approved the study (registration number 2021 − 236) in accordance with the Declaration of Helsinki. Prior inclusion, informed consent was obtained from all participants or their legal guardians. The study was registered at clinicaltrials (NCT04913298).

*Study population*: Adult patients at the ICU with a total bilirubin > 10 mg/dl and the necessity of continuous kidney replacement therapy (CKRT) due to an acute kidney injury (AKI) grade 2 or 3 diagnosed by the KDIGO consensus criteria. CKRT was processed with the Fresenius MultiFiltrate Ultraflux AV 1000 S dialyzer using continuous veno-venous haemodialysis (CVVHD) or continuous veno-venous haemodiafiltration (CVVHDF), depending on the patient’s needs. Patients on CVVHD were anticoagulated with citrate (Fresenius CiCa), while patients on CVVHDF were substituted with Fresenius MultiBic without additional anticoagulation due to impaired liver function. All patients received a CS therapy to support liver excretory function. The adsorber was installed in the extracorporeal circuit downstream of the dialysis filter. Patients without written consent and prior CS therapy were excluded.

### *Data collection*

For data evaluation, demographic data, clinical and laboratory variables were collected from the laboratory and patient information system. Laboratory variables were measured with validated laboratory methods in the Institute of Laboratory Medicine. BA profiling was performed by isotope dilution liquid chromatography-tandem mass spectrometry (LC-MS/MS) with the Biocrates Bile Acids Kit (Biocrates, Innsbruck, Austria) on an acquity ultra-high performance LC system interconnected with a Xevo TQ-S MS/MS (Waters, Milford, MA, USA). Samples with bile acid concentrations exceeding the highest calibrator were diluted with phosphate-buffered saline pH 7.4, re-assayed and concentrations calculated back. Measured TBAs were taurocholic acid, glycocholic acid, taurochenodeoxycholic acid and glycochenodeoxycholic acid,

*Statistical analysis*: Statistical analysis was performed with IBM SPSS statistics (Version 26.0. IBM Corp., Armonk, NY, USA). After examination of a non-normal distribution of some examined parameters (*Shapiro-Wilk test*), *Spearman’s correlation* was performed to analyse TBA and total bilirubin concentrations before and during initiation of CS. *Wilcoxon-test* and *Mann-Whitney-U-test* were used to compare the bilirubin and TBA concentrations before and during CS application. Relative reduction (RR) of bilirubin and TBA was calculated using the formula $\:RR\:\left(\%\right)=\left(\frac{concentration\left(pre-post\right)}{concentration\left(pre\right)}\right)*100$.

## Results

### Demographic and clinical data

In total, 16 patients were included in the evaluation. The reasons for the cholestatic liver dysfunction were in descending order: multiorgan failure (6 patients), liver graft failure (4), sepsis (2), secondary sclerosing cholangitis (2), acute on chronic liver failure (1), and acute liver failure (1), respectively. All patients were treated with ursodeoxycholic acid (250 mg four times a day). The median age was 53 years and 70% were male. The SAPS II (Simplified Acute Physiology score II ) on the day of the CS treatment was 80 and the 28-day mortality was 37.5%. The median MELD Score (Model for the endstage of liver disease) was 34. Detailed patient characteristics, laboratory data especially liver function tests (thrombocytes,, alanine aminotransaminase (ALT), aspartate transaminase (AST), γ-glutamyl transferase (γ-GT)) before and after CS treatment and information about the CKRT can be found in Table 1.

**Table 1:** Patient characteristics, kidney replacement therapy and laboratory data.

	*n* (%) or median [IQR]
*Patient characteristics*	
Age (years)	53 [38, 62]
Gender: male/female	10 (70) / 6 (30)
Weight (kg)	79 [64, 85]
Height (m)	1.69 [1.65, 1.80]
28-days mortality	9 (45)
SAPS II on the study day	80 [69, 90]
MELD	34 [31; 36]
*Kidney replacement therapy*	
Dialyzer	Fresenius MultiFiltrate circuit (MultiFiltrate Ultraflux AV 1000 S).
CVVHD (CiCa)/ CVVHDF (MultiBic, post-dilution)	13 (81) / 3 (19)
Blood flow (ml/min)	100 [100, 128]
Dialysate flow (ml/h)	2000 [2000, 2125]

**Laboratory data**	**Before CS **	**After CS**
Thrombocytes (G/l)	93 [52, 129]	73 [42, 106]
INR	1.1 [1.3, 1.7]	1.5 [1.3, 1.9]
ALT (U/l)	184 [109, 271]	152 [95, 367]
AST (U/l)	180 [112, 677]	172 [100, 728]
γ-GT (U/l)	290 [58, 869]	155 [48, 800]

Note: SAPS II Simplified Acute Physiology score II, MELD Model for the endstage of liver disease, CVVHD continuous veno-venous hemodialysis, CVVHDF continuous veno-venous hemodiafiltration, INR international normalized ratio, ALT alanine aminotransaminase, AST aspartate transaminase, γ-GT γ-glutamyl Transferase.

## Correlation of bilirubin and TBAs before and during CS therapy

**Fig. 1:**
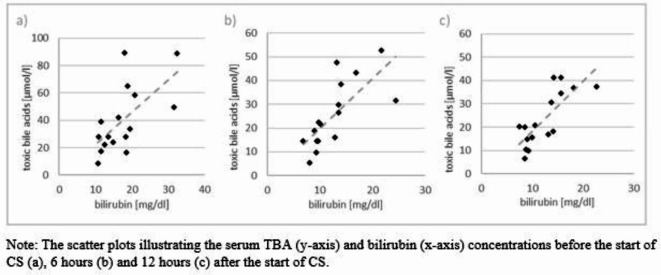
Correlation of bilirubin and toxic bile acids serum concentrations.

A moderate correlation between bilirubin and TBAs was observed before CS therapy (r_start_=0.64, *p* = 0.008) and after 12 h of application (r_12h_ = 0.72, *p* = 0.002). After 6 h of CS application both parameters highly correlated with each other (r_6h_ = 0.85, *p* < 0.001)^8^. Figure 1 shows the different concentrations for each patient at start (a), after six (b) and 12 h (c) in scatter plots.

### Change of bilirubin and toxic bile acids during CS therapy

**Fig. 2:**
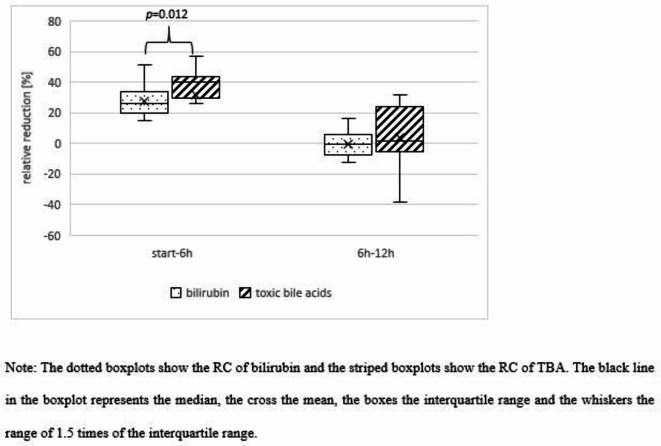
Relative reduction [%] of bilirubin and toxic bile acids due to the adsorber CytoSorb.

At the commencement of CS therapy, the median serum bilirubin concentration was 17.1 mg/dl (IQR: 12.4, 19.0). After 6 and 12 h post-initiation, the median bilirubin concentrations were 11.5 mg/dl (9.4, 13.6) and 11.9 mg/dl (9.0, 14.6), respectively. A significant reduction in bilirubin was observed within the initial 6 h (*p* < 0.001), but no significant decrease in the subsequent 6 h (*p* = 0.918). At the start of CS therapy, the median serum TBA concentration was 30.8 µmol/l (IQR: 23.4, 51.7; CV = 0.59). The median TBA concentrations at 6 and 12 h after CS initiation were 21.9 µmol/l (14.9, 33.3; CV = 0.53) and 20.1 µmol/l (15.4, 35.1; CV = 0.48), respectively. Analogous to bilirubin, a pronounced decrease in TBA was recorded in the initial 6 h (*p* < 0.001), but not in the following 6 h (*p* = 0.115). The median RR of bilirubin between baseline and 6 h was 26.1% (IQR: 19.5,33.4) and from 6 to 12 h, it was − 0.6% (− 5.8, − 6.8). For TBAs, the median RRs were 39.8% (IQR: 31.5, 42.9) and 1.8% (IQR: − 5.2, 16.6) in the respective intervals. Both substances experienced a significant decline in RR during CS application (*p*_*bilirubin*_<0.001, *p*_*TBA*_=0.011). A significant difference in RR between bilirubin and TBA was observed during the first 6 h (start − 6 h: *p* = 0.012), but not in the subsequent period (6–12 h: *p* = 0.445). Figure 2 provides a comparative visual representation of these changes.

## Discussion

Patients in intensive care often have pathological liver function tests for a variety of reasons^9^. Clinically, bilirubin is a commonly referenced parameter for therapeutic choices and progress monitoring^10^, with its prognostic value evidenced in several studies^11^. Concurrently, elevated TBAs are observed in cholestatic liver dysfunction patients^12^, indicating hepatocyte damage^13^. Horvatits et al. identified a higher TBA proportion in critically ill patients than in control groups^2^, suggesting TBAs as potential prognostic indicators in such patients.

Unlike bilirubin, measurement of TBA is limited in many hospitals. Therefore, we wanted to address the important question whether there is a correlation between bilirubin and TBA in ICU patients with cholestatic liver dysfunction. We demonstrated a moderate correlation between the two parameters (*r* = 0.64, *p* = 0.008), so that conclusions can be drawn about the TBA concentration based on the bilirubin concentration. In addition, there was a very strong correlation (r6h = 0.85, *p* < 0.001) between bilirubin and TBA during CS application, suggesting that bilirubin could be used as a surrogate parameter for TBA during CS therapy.

In 2018, Voiosu et al. analysed 108 patients with cirrhosis looking for associations between total bile acids and echocardiographic and biochemical markers of cardiac dysfunction. They also correlated total bile acids with bilirubin and found a strong correlation (*r* = 0.78) between the two parameters^14^. These results are consistent with ours, considering a correlation independent of CS treatment and patients characteristics.

Riva et al. compared the CytoSorb with coupled plasma filtration adsorption and their capability to remove bilirubin and total bile acids from patients’ blood. They showed a efficient removal of both substances. In contrast to our findings the removal rate for total bile acids was lower than for bilirubin.

With liver dysfunction still carrying significant mortality risks and limited therapeutic solutions^15^, there is great interest in liver support therapies and extracorporeal elimination of toxins. According to their toxic potential^16^, the removal of TBAs seems to be a potential therapeutic option^17–19^. Recent in-vitro data indicated that CS effectively reduces TBA levels^20^. Furthermore, one case report described a reduction of TBA in the blood during the application of CS^17^. However, broader, prospective studies are still pending.

Our study discerned a significant reduction in both bilirubin and TBA through CS, though the removal rate diminished after 6 h. A significant higher RC for TBAs (*p* = 0.012) was observed, suggesting that relying on bilirubin alone might underestimate TBA elimination. The adsorber contains highly porous polymer beads that preferentially bind hydrophobic substances up to a molecular size of 60 kDa. According to the manufacturer, Cytosorbents, this results in more efficient removal of highly concentrated substances. Despite these properties, the adsorber binds substances non-selectively and the true adsorption spectrum is still the subject of ongoing research. The molecular characteristics of bilirubin and TBA match the properties of the adsorber, but the actual interactions and binding mechanisms are not well enough understood to draw conclusions about the different removal rates of the two substances. Riva et al. compared CytoSorb with coupled plasma filtration adsorption and their ability to remove bilirubin and total bile acids from patient blood^7^. They showed efficient removal of both substances by CS with similar elimination dynamics, but unfortunately no correlation analysis was performed for bilirubin and total bile acids.

Summarising, Horvatits et al.’s findings^2^ are further bolstered by our results, emphasizing the precision of TBA measurements, especially considering possible BA elevation due to UDCA therapy. Further studies are needed in the future to investigate the clinical effect of TBA removal and the reliability of bilirubin as a surrogate parameter in the clinical routine.

## Conclusion

A moderate correlation exists between serum bilirubin and TBA levels in ICU patients with cholestatic liver dysfunction. CS significantly reduced both bilirubin and TBA levels in the first 6 h of application. The course of the bilirubin concentration therefore appears to be suitable for assessing the elimination of toxic bile acids under CS therapy in patients with cholestatic liver dysfunction. However, the clinical benefits of TBA removal were not part of this study and warrant further research.

## Data Availability

All data generated to answer the research question are included in this article.

## Abbreviations

AKI: Acute kidney injury
ALT: Alanine aminotransaminase
AST: Aspartate transaminase
BA: Bile acid
CKRT: Continuous kidney replacement therapy
CVVHD: Continuous veno-venous haemodialysis
CVVHDF: Continuous veno-venous haemodiafiltration
CS: CytoSorb
γ-GT: γ-glutamyl transferase
ICU: Intensive care unit
MELD: Model for the endstage of liver disease
RC: Relative change
SAPS II: Simplified Acute Physiology score II
TBA: Toxic bile acids
